# Brainstem Auditory Evoked Potentials and Visual Potentials in Kawasaki Disease: An Observational Monocentric Study

**DOI:** 10.3389/fped.2020.581780

**Published:** 2020-12-07

**Authors:** Maria Cristina Maggio, Giovanni Corsello, Giuseppe Salvo, Domenico Giuseppe Puma, Rolando Cimaz

**Affiliations:** ^1^University Department PROMISE of Health Promotion Sciences Maternal and Infantile Care, Internal Medicine and Medical Specialities “G. D'Alessandro”, University of Palermo, Palermo, Italy; ^2^Pediatric Neuropsychiatry Operative Unit, Children Hospital “G. Di Cristina”, ARNAS, Palermo, Italy; ^3^Department of Clinical Sciences and Community Health, University of Milan, Milan, Italy

**Keywords:** kawasaki disease, brainstem auditory evoked potentials, visual evoked potentials, coronary artery lesions, intravenous immunoglobulins

## Abstract

**Background:** Kawasaki Disease is a systemic vasculitis, particularly involving coronary arteries. Rare involvement of other vascular districts is described, as central nervous system arteries, leading to a vasculitic neuropathy. Sensorineural hearing loss and alterations of evoked potentials are uncommonly reported complications.

**Methods:** In an observational monocentric study, 59 children (37 males; 22 females; mean age: 2.7 ± 2.2 years) with documented Kawasaki Disease were enrolled. No risk factors for hearing loss and/or neurological impairment were identified in the cohort. Brainstem auditory evoked potentials and visual evoked potentials were correlated with clinical, hamatological and radiological data, evaluated in the acute phase of the Kawasaki Disease, and during the follow-up.

**Results:** Evoked potentials were altered in 39/59 patients (66%): of these, 27/39 (69%) showed altered IV and V waves and/or III-V interwave latencies of brainstem auditory evoked potentials; 4/39 (10%) showed pathological visual evoked potentials; 8/39 (21%) had abnormalities of both brainstem auditory evoked potentials and visual evoked potentials. No permanent deafness was reported.

**Conclusion:** Abnormalities in visual evoked potentials were not significantly correlated with coronary artery lesions; however, the presence of abnormalities of brainstem auditory evoked potentials were associated with the risk of coronary artery lesions.

## Introduction

Coronary artery lesions (CAL) and/or cardiac lesions are the fearsome evolution of Kawasaki disease (KD). Nevertheless, other vascular districts may be involved less frequently, such as central nervous system (CNS) arteries (1, 2), peripheral *vasa nervorum* and perineural blood vessels, leading to a vasculitic neuropathy of acoustic nerve and optic nerve (3, 4).

A severe cochlear or vessel wall inflammation can induce hearing loss and/or subclinical alteration of brainstem auditory evoked potentials (BAEPs). Optic nerve could be involved as well, with a pathological pattern of visual evoked potentials (VEPs). However. these abnormalities are sporadic and have been described just in a few reports (4, 5).

In the literature, only few studies focused on BAEPs and VEPs in KD (6). There are also some case reports of auditory loss secondary to KD (4, 5).

Sensorineural hearing loss may be underreported, since a reduced hearing acuity may be only transient and since when the condition is present in young children it could not be promptly recognized. Hearing loss is often detected only by audiometry or, in young children in whom tone audiometry is difficult to be carried out, by BAEPs.

Some reports showed that up a third of KD children had a sensorineural auditory loss during the acute and subacute phases of the disease, and that the auditory loss could be maintained even six months after diagnosis (7, 8). The authors observed a positive association of this complication with anemia and thrombocytosis. VEPs and BAEPs may be pathological in the acute phase of KD, however they can normalize during the follow-up. This finding may be the sign of systemic vasculitis, involving CNS.

CAL may have poor correlation with clinical signs; however, hematological parameters, expression of critical cytokine secretion (such as leucocytosis, increased neutrophil percentage, elevation of CRP, increased transaminases, hypoalbuminemia and hyponatremia), are correlated with a more severe evolution and an increased risk to develop CAL. Pharmacological approach of KD with IVIG within 7-10 days since KD onset is efficacious to prevent CAL. However, there are no data about hematological parameters and the treatment choice on the prevention of evoked potentials alterations.

A systemic vasculitis could involve many districts at the same time and in the acute phase of the disease; the synchronous involvement of coronaries and CNS districts in KD has not yet been demonstrated.

### Aims of the Study

Aims of the study were:

- the evaluation of VEPs and BAEPs in children affected by KD, in the acute phase of the disease and during the follow-up;
- the evaluation of the role of evoked potentials as an added tool to detect occurrence of vasculitis in the anatomic and functional systems respectively explored by VEPs and BAEPs;
- the correlation of VEPs and BAEPs with clinical signs, hematological parameters, treatment with IVIG, aspirin and other drugs;
- the correlation between CAL and/or cardiac lesions, and dysfunction of VEPs and BAEPs at diagnosis and during the follow up.

## Materials and Methods

### Patients

In an observational monocentric study, we enrolled 59 children (37 M; 22 F; mean age: 2.7 ± 2.2 years; range 3 months−10 years) with documented KD. All the patients, followed from 2012 to 2018 in the Pediatric Clinic of Palermo, Children Hospital “G. Di Cristina,” ARNAS Palermo, with a good compliance to the procedures of evaluation of evoked potentials, and no risk factors for hearing loss and/or neurological impairment (CNS congenital diseases, developmental delay, ASA intoxication, etc.) were included. Of those, 37 children (63%) had typical KD, eight patients (14%) atypical KD, and 14 patients (23%) an incomplete form. All patients were treated with 2 g/kg of intravenous immunoglobulins (IVIG) and acetylsalicylic acid (ASA) at the dosage of 30–50 mg/kg/day in the acute phase, and 3–5 mg/kg /day as antiplatelet prophylaxis. BAEPs and VEPs were correlated with clinical, hematological and radiological data, evaluated in the acute phase of the KD, and during the follow up.

### Clinical and Demographic Data

Age, gender, days of illness at initial treatment and response to IVIG and ASA; rash; changes of lips and oral mucosa; conjunctivitis; cervical lymphadenopathy.

### Hematological Parameters

White blood cell count, neutrophils percentage, platelet count, hemoglobin, C-reactive protein (CRP), erythrocyte sedimentation rate (ESR), transaminases, gamma-glutamyl transferase, ferritin, albumin, sodium, D-dimer.

### Imaging Data

Chest x-ray, echocardiogram, abdominal ultrasound.

### Diagnostic Criteria

KD was diagnosed following the internationally approved criteria (9, 10):

- Typical KD was diagnosed when fever lasting more than 5 days was associated with ≥4 clinical criteria:
  1. bilateral non-exudative conjunctivitis
  2. changes of oral mucosa and lips
  3. changes of the extremities
  4. polymorphous exanthema
  5. cervical lymphadenopathy.
- Incomplete KD was suggested in every infant showing fever lasting more than 5 days with documented systemic inflammation if <4 main clinical features were found after exclusion of many febrile illnesses.
- Atypical KD was diagnosed if fever lasting more than 5 days, not otherwise explained, was associated with non-classic manifestations (as sensorineural hearing loss, aseptic meningitis, seizures, peripheral facial nerve palsy, acute abdomen, pancreatitis, gallbladder hydrops, pneumonia, arthritis, orchitis, renal impairmant, sterile pyuria). CAL documented by echocardiography confirmed the diagnosis.

### Ethics and Ethics Committee

Ethics committee approval according to local regulations was not necessary since this was part of our clinical practice and since deidentified data were used for statistical analysis. However, the study involving human participants was reviewed and approved by the ethics committee Palermo 1 (ARNAS Civico, Palermo, Italy). The patients' legal guardian provided written informed consent to participate in this study. The written consent is in the documents in the hospital “G. Di Cristina”, ARNAS Palermo, Italy.

### Methods

VEPs and BAEPs were recorded by a MYOQUICK SystemPLUS Evolution (Micromed, Italian). The relevant recording techniques are described hereunder.

The latencies evaluation of waves I, III and V were performed in all patients together with the amplitudes of waves I and V. Furthermore, the V/I amplitude ratio, and the I-III and III-V interwave latencies were calculated. Recorded abnormalities consisted of decreased amplitude of wave V and prolonged III-V interpeak latencies.

The recordings were collected in the acute phase of the disease, in the few days (1–7, 9–11) after defervescence occurred, before discharge from the hospital, and during follow-up (6 and 12 months after discharge).

### VEPs

VEPs were recorded from an active midline occipital electrode over the visual cortex at O_z_, with a midline frontal reference electrode at F_z_, according to the Recommendations and Guidelines of the International Federation of Clinical Neurophysiology (IFCN) Committee (12) and International Society for Clinical Electrophysiology of Vision (ISCEV) (13). On the basis of children's age and of his/her degree of cooperation during the exam, different types of stimulus have been used.

**Flash VEPs** were performed with monocular stimulation. The unstimulated eye was occluded to avoid extraneous and unwanted stimulation. The potentials were carried out using a flash (brief luminance increment) with a stimulation force of 3 cd s m^−2^ (photopic candles seconds per square meter) which subtends a visual field of at least 20° in a dimly illuminated room.

- **Pattern Reversal Visual Evoked Potentials (PRVEPs)** were performed with monocular stimulation and full-field stimulation.

The standard stimulus for VEPs is a checkerboard model in which the squares take turns from black to white: the dark squares become light and vice versa without changing the general luminance of the display.

The pattern was reversed 100 times at 1 Hz and the results were then averaged; A repeated trial of averaged stimuli was also recorded.

### BAEPs

The BAEPS were carried out and interpreted according to the guidelines of the American Clinical Neurophysiology Society (ACNS) (14).

BAEPs were recorded from an active electrode positioned over Cz and referred to the right (M2) and to the left (M1) mastoid process. Waveforms are recorded from ipsilateral and contralateral pathways simultaneously, allowing easier recognition of individual peaks.

The following regards others technical details:

- the stimuli consisted of 100/microseconds clicks delivered to each ear in turn through pre-calibrated shielded earphones at a rate of 10.6 Hz.
- the stimulus intensity was 60 dB above the hearing threshold (14) for each individual ear with the contralateral ear masked by white noise of 40 dB below the stimulus intensity;
- Thousand and five hundred or more stimuli, depending on the shape and amplitude of the potentials, were averaged;
- the latencies of waves I, III, and V were measured together with the amplitudes of waves I and V. The V/I amplitude ratio, and the I-III and III-V interweaves latencies were also calculated.

### Statistics

Patients were classified by gender, age, treatment duration and evoked potentials normal or altered. Correlation between the analyzed parameters collected were performed using Chi-square test. All variables were tested for normal distribution using the Anderson-Darling normality test. All ordinal data were expressed as numbers and percentages. Calculations were done using “MiniTAB release 13.1 Statistical Software.”

## Results

### Evoked Potentials Abnormalities

The data of evoked potentials findings for all the patients, divided in three groups of KD (typical, atypical, incomplete), are reported in Table 1.

**Table 1:** BAEPs and VEPs findings (normal or pathological) were divided in three groups of KD (typical, atypical and incomplete KD).

	**Patients *n* = 59**	**Normal BAEPs and VEPs 20/59 (34%)**	**Pathological BAEPs only 27/59 (45%)**	**Pathological VEPs only 4/59 (7%)**	**Pathological BAEPs and VEPs 8/59 (14%)**
Typical KD	37/59 (63%)	11/37 (30%)	19/37 (51%)	1/37 (3%)	6/37 (16%)
Atypical KD	8/59 (14%)	4/8 (50%)	2/8 (25%)	2/8 (25%)	0
Incomplete KD	14/59 (23%)	5/14 (36%)	6/14 (43%)	1/14 (7%)	2/14 (14%)

Evoked potentials were altered in 39/59 patients (66%) (26 M; 13 F): among these, 27/39 (69%) (18 M; 9 F) had pathological BAEPs; 4/39 (10%) (2 M; 2 F) had pathological VEPs; 8/39 (21%) (6 M; 2 F) had abnormalities of both BAEPs and VEPs. Hence, BAEPs were altered in 35 patients (24 M; 11 F) (Table 2).

**Table 2:** BAEPs and VEPs findings in KD patients: 26/37 (70%) of males and 13/22 (59%) of females showed pathological evoked potentials.

	**Patients with pathological evoked potentials *n* = 39**	**Pathological BAEPs only 27/39 (69%)**	**Pathological VEPs only 4/39 (10%)**	**Pathological BAEPs and VEPs 8/39 (21%)**
Males (37)	26 (67%)	18 (67%)	2 (50%)	6 (75%)
Females (22)	13 (33%)	9 (33%)	2 (50%)	2 (25%)

Pathological BAEPs waves were documented as bilateral in most of the patients. However, 12 patients (31%) showed unilateral pathological BAEPs.

In two patients hearing loss was documented, and one of them showed neurosensorial hypoacusis. This was a 4 month-old baby, who had complete KD with CAL, in whom hypoacusis was found after the resolution of the acute phase and was associated with pathological VEPs: at the following controls, VEPs were still pathological, while BAEPs were in the normal range and hearing loss had resolved. He was treated with IVIG 8 days after KD onset and did not receive steroids.

In the patients with VEP abnormalities, the prevalent recorded features were the increased latency of P100 /P2 wave-Flash VEP (P2) or the pattern VEP (P100).

There was no significant difference in incidence of evoked potentials alterations between typical or incomplete KD patients.

### Cardiac Involvement

In our patients, CAL were detected in 17/59 patients (29%): six children (10%) showed coronaritis without aneurysms, 11 (19%) developed aneurysms. 6/17 children with CAL had normal BAEPs and VEPs; 4/17 children showed both altered BAEPs and VEPs; 6/17 patients had altered BAEPs; 1/17 patients had altered VEPs (Table 3).

**Table 3:** Pathological BAEPs and VEPs and CAL: distribution of the cases.

	**Patients *n* = 59**	**Normal BAEPs and VEPs 20/59 (34%)**	**Pathological BAEPs only 27/59 (46%)**	**Pathological VEPs only 4/59 (7%)**	**Pathological BAEPs and VEPs 8/59 (14%)**
No CAL	42 (71%)	14/42 (33%)	21/42 (50%)	3/42 (7%)	4/42 (10%)
CAL	17 (29%)	6/17 (35%)	6/17 (35%)	1/17 (6%)	4/17 (24%)

The incidence of pathological BAEPs and VEPs was higher than CAL; in fact, 71% of the patients had normal echocardiogram, while only 34% of the patients showed normal evoked potentials. Nevertheless, 50% of children with both pathological BAEPs and VEPs had CAL; 35% of patients with pathological BAEPs and normal VEPs showed CAL; 6% of patients with pathological VEPs and normal BAEPs had CAL.

In some patients of our case series, evoked potentials alterations appeared earlier than CAL, while in two patients these alterations were detected only during follow-up (at 12 months after disease onset) (Table 4).

**Table 4:** BAEPs and VEPs during the acute phase and 12 months after the onset of the disease.

**Patients with pathological evoked potentials**	**Patients with pathological evoked potentials normalized during the follow-up**	**Patients with normal evoked potentials pathological during the follow-up**
39	3	2

### Correlation With Clinical and Hematologic Parameters

No correlation was seen between BAEPs and VEPs alterations and days of fever at the start of IVIG, pre-IVIG D-Dimer plasma levels, CRP, ESR, leukocyte count, neutrophil percentage pre- and post- IVIG, number of IVIG doses.

### Correlation With Treatment Strategies

**Figure 1:**
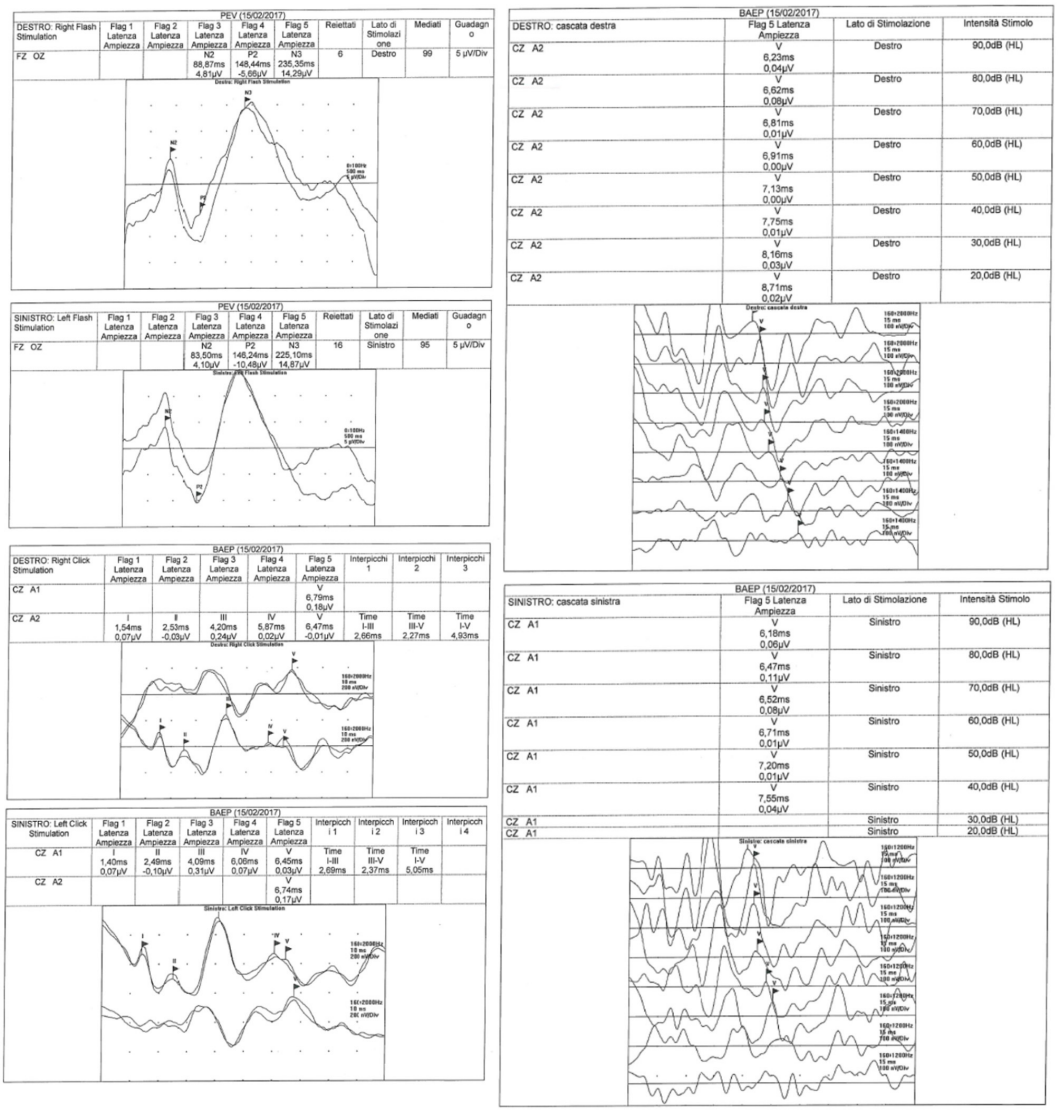
VEPs and BAEPs in a patient with pathological records.

Furthermore, pathological evoked potentials and the resolution of these abnormalities in 3/39 (8%) patients during follow-up, were not correlated with corticosteroid treatment (which was administered in eight patients), while treatment with anakinra induced the resolution of CAL, as documented in the literature (15, 16) and the normalization of evoked potentials in one child (Figure 1).

## Discussion

The patients studied in our series showed altered IV, V waves and/or III-V interwave latencies, despite normal hearing acuity. These children showed a possible CNS vasculitis, and in a high percentage of them CAL were also present. Recorded abnormalities of BAEPs consisted of decreased amplitude of wave V, prolonged III-V interpeak latencies caused by slowed conduction within the lower pons and the midbrain (17).

In this case series evoked potentials were pathological in a high number of patients: 70% of males and 59% of females. BAEPs, alone or in association with VEPs, were more frequently pathological than VEPs alone. In fact, isolated alteration of VEPs was found in only 7% of the patients. Otherwise, isolated alterations of BAEPs were detected in 46% of the patients, and most of them showed alterations secondary to mesencephalic involvement.

Despite normal hearing acuity in almost all cases, the patients of our cohort showed a neuronal involvement, documented by evoked potentials. The dysfunction of our patients seemed secondary to a mesencephalic damage. This pattern could be an expression of a vasculitis of CNS, which has already been documented in KD children in other encephalic districts (1, 2, 8). In 43% of these children CAL were present, possibly as further expression of systemic vasculitis.

The abnormalities of BAEPs and VEPs are not necessarily linked to the acute phase of KD; in fact, these alterations have been documented also months after KD onset, and some patients may maintain a permanent loss of function with an auditory loss (3, 5, 7, 18–20). The spectrum of complications in KD includes not only CAL and other cardiac lesions, but also CNS involvement.

In our study, the percentage of children with pathological BAEPs and/or VEPs in association with CAL is high. However, the differences between the groups of patients with specific evoked potentials alterations did not reach the statistical significance, but the number of patients in the specific groups are small and need further confirmation in a larger population sample. Moreover, our study of CNS involvement was performed by evoked potentials, suggesting a functional involvement of the encephalic districts, but was not confirmed by Magnetic Resonance Angiography. Further studies could be integrated with Magnetic Resonance Angiography in children with KD, to document anatomic damage secondary to systemic vasculitis.

A multicentre report on KD patients showed transient sensorineural hearing loss as a possible complication of acute KD; this event was considered secondary to salicylate toxicity (21). A persistent sensorineural hearing loss is rare (19). High doses of salicylates could cause severe hearing loss; however, in our series all patients were treated with low-medium doses of ASA. Case reports described bilateral severe sensorineural hearing loss, in some cases treated with corticosteroids; some experienced only a partial improvement, with possible severe persisting hearing loss (18, 19).

An association between persistent sensorineural hearing loss and persistent thrombocytosis, anemia, high ESR and late administration of IVIG has been described (7); in our case series this association was not found, neither with hematological parameters nor with clinical signs. Furthermore, IVIG, steroids and ASA timing of administration was not correlated with evoked potentials alterations. Hence, the finding of evoked potentials abnormalities did not modify the treatment of our patients; however, they can help the clinicians in the diagnosis, especially in atypical or incomplete cases. In this regard, treatment can be guided by BAEPs and VEPs study, in terms of an earlier diagnostic suspicion and a prompt therapeutic choice.

A generalized vasculitis, involving CNS vessels, was documented in KD (2, 22), describing hypoperfusion events that occur in the acute phase of KD, and provides another physio-pathological hypothesis that supports potentials alteration. In fact, as further confirmation of the hypothesis of the systemic vasculitis, in our series several patients showed CAL as well. We could not demonstrate a statistically significant association between abnormal potential and CAL, but the limited number of children of each group did not contribute to achieve a statistically significant difference.

## Conclusion

In conclusion, sensorineural hearing loss and alterations of evoked potentials are uncommonly reported complications of KD. In the present sample of patients, CNS involvement was always subclinical, without symptoms otherwise reported by patients and/or parents. These data alert the clinicians about the meaning of pre-clinical evoked potentials abnormalities in the management of KD patients. We hypothesize that vasculitis of vasa nervorum is a possible pathogenetic event linked to evoked potentials alterations. Evoked potentials allow to detect brain dysfunction, supporting brain involvement and precociously helping to identify permanent lesions that can induce disability if underdiagnosed. Furthermore, the diagnosis of atypical or incomplete KD is further supported by evoked potentials study.

A few studies proposed that asymptomatic cerebral vasculitis might be more frequent than expected on the basis of clinical signs (23). Therefore, pediatricians must consider the possible vascular involvement beyond CAL in KD, especially in the cerebral vessels, for the life-treating consequences and the poor prognosis of these patients.

## Data Availability Statement

The raw data supporting the conclusions of this article will be made available by the authors, without undue reservation.

## Ethics Statement

The study involving human participants was reviewed and approved by the ethics committee Palermo 1 (ARNAS Civico, Palermo, Italy. The patients' legal guardian provided written informed consent to participate in this study.

## Author Contributions

MM, GS, and DP: substantial contributions to conception and design, acquisition of the data, analysis and interpretation of the data. RC and GC: drafted the article and revised it critically for important intellectual content. MM, GC, GS, DP, and RC: final approval of the version to be published. All named authors have agreed to its submission.

## Conflict of Interest

The authors declare that the research was conducted in the absence of any commercial or financial relationships that could be construed as a potential conflict of interest.

## Abbreviations

KD: Kawasaki Disease
BAEPs: brainstem auditory evoked potentials
VEPs: visual evoked potentials
CAL: coronary artery lesions
CRP: C-reactive protein
ESR: Erythrocyte sedimentation rate.
